# Feature discrimination learning transfers to noisy displays in complex stimuli

**DOI:** 10.3389/fcogn.2024.1349505

**Published:** 2024-03-06

**Authors:** Orly Azulai, Lilach Shalev, Carmel Mevorach

**Affiliations:** ^1^School of Education, Tel Aviv University, Tel Aviv, Israel; ^2^The Sagol School of Neuroscience, Tel Aviv University, Tel Aviv, Israel; ^3^School of Psychology, University of Birmingham, Birmingham, United Kingdom; ^4^Centre for Human Brain Health, University of Birmingham, Birmingham, United Kingdom

**Keywords:** perceptual learning, learning transfer, specificity, face stimuli, complex stimuli, signal in noise, feature discrimination

## Abstract

**Introduction:**

Perception under noisy conditions requires not only feature identification but also a process whereby target features are selected and noise is filtered out (e.g., when identifying an animal hiding in the savannah). Interestingly, previous perceptual learning studies demonstrated the utility of training feature representation (without noise) for improving discrimination under noisy conditions. Furthermore, learning to filter out noise also appears to transfer to other perceptual task under similar noisy conditions. However, such learning transfer effects were thus far demonstrated predominantly in simple stimuli. Here we sought to explore whether similar learning transfer can be observed with complex real-world stimuli.

**Methods:**

We assessed the feature-to-noise transfer effect by using complex stimuli of human faces. We first examined participants' performance on a face-noise task following either training in the same task, or in a different face-feature task. Second, we assessed the transfer effect across different noise tasks defined by stimulus complexity, simple stimuli (Gabor) and complex stimuli (faces).

**Results:**

We found a clear learning transfer effect in the face-noise task following learning of face features. In contrast, we did not find transfer effect across the different noise tasks (from Gabor-noise to face-noise).

**Conclusion:**

These results extend previous findings regarding transfer of feature learning to noisy conditions using real-life stimuli.

## 1 Introduction

The term perceptual learning refers to change in performance (often improvement) of perceptual tasks resulting from training or practice, which includes repeated exposure to a specific stimulus (Fahle, 2005; Gold and Watanabe, 2010). Perceptual learning can improve detection, discrimination, or identification of visual stimuli. These improvements usually manifest in increased accuracy or better ability to perform a task with more difficult stimulus (e.g., lower contrast or shorter durations) at a critical and given level of accuracy (Dosher and Lu, 2005). Conventional paradigms of perceptual learning research maintain the concept of learning being often specific to retinal location or to a stimulus feature such as orientation, spatial frequency or scale (e.g., Karni and Sagi, 1991; Polat and Sagi, 1994; Crist et al., 1997). Nevertheless, generalization (transfer) of perceptual skills has also been observed (e.g., Dosher and Lu, 2005; Bi et al., 2010; Chang et al., 2013). While the circumstances in which transfer occurs are still subject to debate, studies in perceptual learning emphasize that specificity or transfer are related to a number of factors, including: task difficulty (Ahissar and Hochstein, 1997), number of trials (Aberg et al., 2009), training protocols (Xiao et al., 2008), and task precision (Jeter et al., 2009).

One interesting phenomenon in the context of transfer in perceptual learning is the existence of an asymmetry of transfer after training on perceptual tasks. In a first example, Dosher and Lu (2005) demonstrated such asymmetry, where training on a Gabor orientation discrimination task in the periphery resulted in reduced contrast thresholds for similar Gabor stimuli when combined with white, random Gaussian noise, but not vice versa. Thus, training with “clear” stimuli (without added external noise) improved performance when external noise was added to the same stimuli, but training with “noisy” stimuli (embedded in external noise) did not improve perception of the same stimuli when the external noise was removed. Later studies demonstrated that this asymmetric transfer is stable across different perceptual tasks (Chang et al., 2013). Specifically, Chang et al. (2013) showed that training on a feature discrimination task with “clear” stimuli improves participant's performance on a coarse discrimination judgement when external noise is added to the stimuli. This transfer effect was demonstrated in tasks requiring depth, motion and orientation judgment using random dot stereogram (Chang et al., 2013). However, transfer from the “noisy” task condition to the “clear” feature discrimination task condition was considerably more limited. Such studies highlight both stimulus specific changes in perception and changes to external noise filtering mechanism that follow training (e.g., Dosher and Lu, 1998, 1999; Gold et al., 1999).

These results may indicate that effective performance of tasks with a noise component rely on two functions: Segmentation of the visual feature (or noise filtering) and feature representation (Dosher and Lu, 2005; Chang et al., 2013), while performance on tasks with clear (no noise) stimuli relies only on feature representation. Thus, training on feature representation can transfer to tasks with noise. Interestingly, Chang et al. (2013) also demonstrated transfer of learning across different noise tasks requiring different perceptual processes such as depth, motion, and orientation. This finding points to the potential generality of the segmentation/noise filtering component that noise tasks rely on, which is called upon across different perceptual dimensions. Consequently, feature representation training promotes feature templates that are important for both tasks (external noise task and feature difference task) with the same feature dimension. Noise task training promotes a general process of noise filtering, independently of stimulus properties (Chang et al., 2013).

While most traditional perceptual learning studies in adults focused on elementary visual features, including orientation (Schoups et al., 1995; Crist et al., 1997; Matthews et al., 1999), patterns and texture (Karni and Sagi, 1991), and other stimulus features, some studies documented perceptual learning with more complex visual stimuli (e.g., Gold et al., 1999; Furmanski and Engel, 2000; Sigman and Gilbert, 2000; Baeck and Op de Beeck, 2010; Bi et al., 2010). In particular, several psychophysical studies reported perceptual learning effects using face stimuli, including a face recognition task (Gold et al., 1999); a face view discrimination task (Bi et al., 2010); and a facial expression recognition task (Du et al., 2016; Russo-Ponsaran et al., 2016). Aligned with the effects of perceptual learning with simple visual features, the effect of perceptual learning with complex stimuli (i.e., real world objects or faces that incorporate many features together into a whole identifiable percept) appears specific to the trained feature (Sigman and Gilbert, 2000), but in several studies a transfer effect from one task to another was also apparent (objects: Furmanski and Engel, 2000; faces: Bi et al., 2010; Du et al., 2016). For instance, Bi et al. (2010) measured learning and transfer processes following face-view training. In their first experiment, participants were trained to discriminate face views around the face orientation of 30°. Two face views were presented successively and participants were asked to judge whether the second face was tilted to the right or to the left relative to the first one. In the pre- and post-training session, face views discrimination thresholds were measured at the face orientations of −90°, −60°, −30°, 0°, +30°, +60°, +90°. The results showed that perceptual learning was specific for the trained face view orientation. However, in additional experiments following a similar protocol the authors report transfer of learning across changes in face size (the test stimuli were bigger in size) and location (upper and lower visual fields).

The separate mechanisms of segregation (noise filtering) and feature representation and how they are trained and transferred in the context of complex visual stimuli has not been investigated thus far. Furthermore, while there is initial evidence pointing to the possible generalizability of noise filtering learning (Chang et al., 2013), the boundaries of this effect are still not completely clear. For instance, is it the case that noise filtering can transfer from simple to complex stimuli when both are embedded in similar noise? To our knowledge, there are currently no experimental results showing transfer effect from “clear”/features training to “noisy” tasks using face stimuli, as well as transfer of noise filtering from simple feature noise task to a face-noise task. If transfer effects from “clear”/features training to noisy tasks can be replicated within complex stimuli it may have implications for developing effective rehabilitation protocols with real-life visual input for those who suffer from noise filtering deficit (i.e., older adults or individuals with Attention-Deficit/Hyperactivity Disorder).

Consequently, the main aim of the present study was to examine the transfer of learning of feature representation to noise tasks using complex visual stimuli (i.e., faces). We ask whether the feature-to-noise transfer effect, which has been demonstrated using simple stimuli, can also be demonstrated in complex real-life images of faces. Based on previously reported similarities in perceptual learning between simple and complex stimuli, we expect to find such a feature-to-noise transfer using our face stimuli. More specifically we expect to find an improvement in a face discrimination task when the face is embedded in noise (face-noise task), following both training in the same task and training in a face discrimination task without noise (face-feature task).

A second, exploratory, aim of the current study was to examine noise task generalization between simple and complex visual features. Previous research found a generalized process of noise filtering mechanism across tasks involving simple visual stimuli and with similar characteristics input of the visual features. Thus, here we ask whether noise task generalization may be found even across stimulus complexity. Specifically, we assessed if training with a simple noise task (using a classical orientation discrimination task using Gabor patches embedded in noise) transfers to a face discrimination task when the faces are embedded in a similar noise (the face-noise task). This enabled us to examine whether the noise filtering mechanism is completely independent of the visual feature, as long as the noise itself remains similar.

## 2 Materials and methods

### 2.1 Participants

Fifty-five students were recruited as participants for the study and were all naïve to the purpose of the study and had never participated in a perceptual learning experiment before. Participants were excluded from further participation in the study and analysis if they could not discriminate between the target faces (nine participants) or if they failed to show perceptual learning (nine participants). Detailed description of these exclusions steps is included below (Sections 2.5 and 2.6, respectively). Thus, thirty-seven participants were included in the analyses (mean age: 24.9 years, age range: 18–35). All were paid for their participation in the experiment, had normal or corrected-to-normal visual acuity as measured by the ETDRS acuity chart, and had no neurological or vision disorders (self-report). All participants gave their written and informed consent in accordance with the protocol approved by the ethics committee of Tel Aviv University (ethics protocol no: 10192340_20170315).

**Figure 1:**
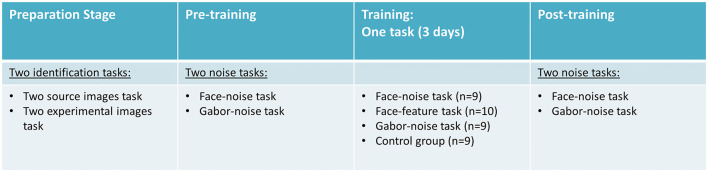
The general protocol of the different training tasks. Participants performed the two noise tasks (face and Gabor) before and after the training they were assigned to. The study included three active perceptual learning training groups and a passive control group.

Participants were equally and randomly divided into four groups. All groups performed the same perceptual tasks before and after 3 days of training, but each group used a different training task in those 3 days (see Figure 1): (1) nine participants trained on face-noise task (six females); (2) 10 participants trained on face-feature task (eight females); (3) nine participants trained on Gabor-noise task (six females). Finally, we also included (4) a control group of nine participants (eight females), who did not perform psychophysical training during the three training days, and only attended the pre-training and post-training sessions, in order to monitor effects attributable to familiarity with the tasks.

### 2.2 Apparatus and display

The stimuli were presented on a 23-inch. Samsung LED monitor at a resolution of 1,920 × 1,080 pixels with a refresh rate of 120 Hz. The display was gamma corrected. All stimuli were presented on a mid-gray background. Custom experimental software was written in PsychoPy2 Experiment Builder (version 1.81.0). The participants' head position was stabilized using a chin rest at a viewing distance of 50 cm, and the experiment was performed in a dark room, in which the only light source was the monitor.

### 2.3 Stimuli and tasks

#### 2.3.1 Face tasks

##### 2.3.1.1 General faces stimuli

**Figure 2:**
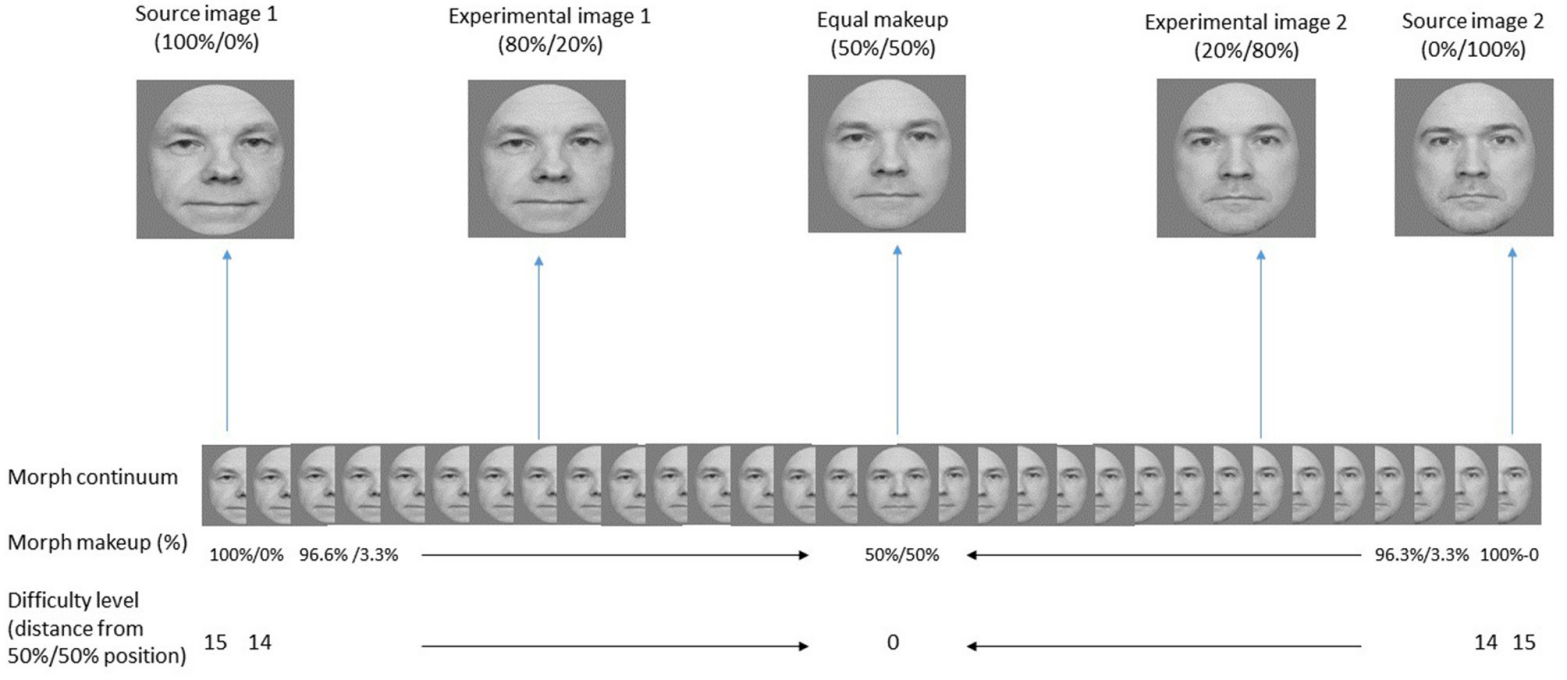
The morphed faces used in the face-noise and face-feature tasks. The full set of the possible morphs (Morph continuum) is presented in the middle of the figure. The two source images and the two experimental images are presented separately at the top. Also presented there is the 50%/50% morph that represents a morph with equal weights of the two source images (i.e., an image that cannot be identified as one identity rather than the other). The weighting of the morphs appears in percentage (Morph makeup). The numbering at the bottom row depict the distance from the midline in morph steps. The distance is given in numbers from 0 (50%/50% morph) to 15 (100%/0% or 0%/100% morphs) in both directions. Larger numbers (distances) mean easier identity identification. Overall, there were 31 face images. Note the actual faces used in the study are not available for publication and were replaced here with similar images.

One pair of Caucasian men (referred to as source images) was selected from the Stirling/ESRC 3D Face Database (http://pics.stir.ac.uk/ESRC/). These two faces, source image 1 and source image 2, were grayscale and scaled to 270 × 270 pixels (Figure 2). Using a morphing program, Morpheus Photo Mixer, we located manually 244 points (especially on facial features such as eyes, nose and mouth) on every source image and created 29 intermediate morph faces between them in steps of 3.3% from 100/0% to 0/100% (see Figure 2 for demonstration of the morph sequence).

##### 2.3.1.2 Face-noise task

###### 2.3.1.2.1 Stimuli

For the face-noise task only two exemplar images from the list of 31 images were used. These were image 7 and image 25 which represented an 80/20% combination of the two source faces. The images were 3° × 4° of visual angles and appeared centrally on every trial. These images were embedded in additive Gaussian noise of mean value 0 and SD of 0.33.

**Figure 3:**
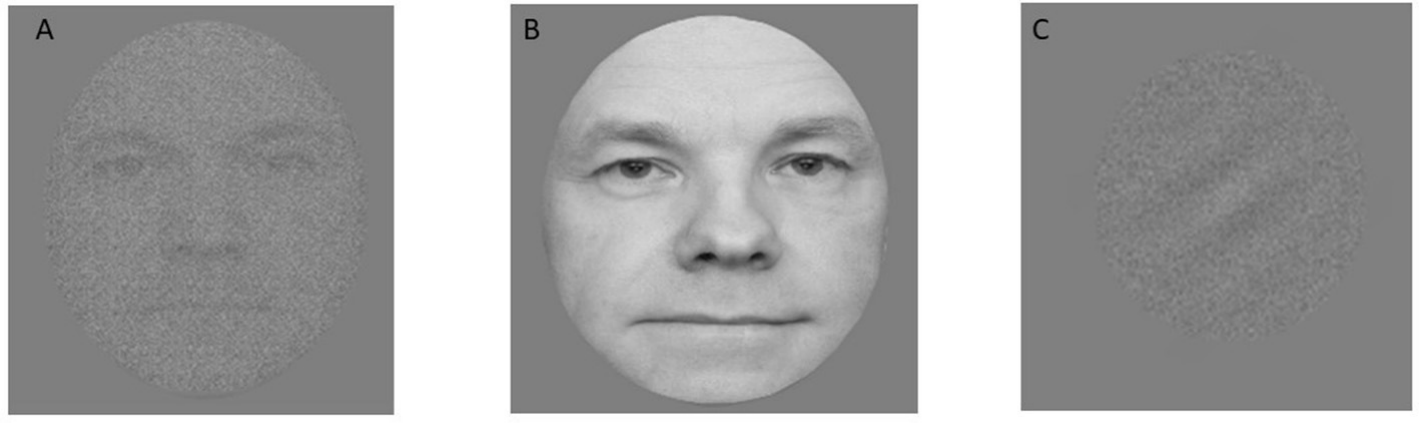
Illustration of stimuli from the three training tasks: **(A)** face-noise task; **(B)** face-feature task; and **(C)** Gabor-noise task. Note the actual faces used in the study are not available for publication and were replaced here with similar images.

The observed noise thresholds on this face-noise task were varied trial-by-trial according to participants' responses and estimated using a staircase procedure (see Figure 3). We manipulated the observed noise by changing the opacity of the noise component. The value of the opacity ranged from 0 (invisible) to 0.99 (opaque). Thus, higher opacity level translates to higher observed noise. The staircase was set up to ensure a reduction of the noise opacity after each error and an increase of the noise opacity after three successive correct responses (a 3/1 staircase). Threshold estimates were then depicted as 1—opacity. Thus, lower thresholds (i.e., 1—opacity of the noise) represent higher levels of noise opacity (higher noise intensity observed on the face stimuli). The opacity of the face stimuli was kept constant at 0.2 throughout. Weber Contrast is 0.14.

###### 2.3.1.2.2 Procedure

In the face-noise task, participants were required to dissociate between two experimental images. Each trial began with a presentation of a small black central point (0.2° of visual angle in diameter) for 250 ms, followed by a blank interval of 300 ms. Afterwards, a target single face stimulus appeared at the center of the screen for 66.64 ms. The inter-trial interval (ITI), from response to the presentation of the fixation point, was 900 ms. Participants were instructed to identify the faces by response keys on the keyboard: left arrow key for experimental image 1 and right arrow key for experimental image 2. Participants were required to respond as accurately as possible at a comfortable speed.

In the pre/post testing the face-noise task was comprised of three blocks whereas the training tasks (face-noise/face-feature/Gabor-noise) included five blocks. Overall, in each block, trials were presented in two randomly interleaved staircases (Levitt, 1971), with different noise opacity starting points: 0.85 and 0.75 (i.e., 1—opacity of the noise) representing low and high discrimination thresholds, respectively. In each staircase, step sizes were progressively adjusted—the step size was 2 dB for the first two reversals, 0.8 dB for the next three reversals and 0.4 dB for the remaining reversals. Each block was completed after at least 120 trials (60 trials for each staircase) and at least six reversals for each staircase occurred. Note that the up and down procedure was 1 dB until the first reversal was reached. This procedure ensures a threshold estimate equivalent to 80% correct performance. On each staircase within an experimental block, we excluded the first three reversals and calculated the discrimination threshold as the average of all remaining reversals. The overall estimated discrimination threshold for the run was averaged across all staircases across all blocks.

##### 2.3.1.3 Face-feature task

###### 2.3.1.3.1 Stimuli

For the face-feature (fine discrimination) task we used the entire set of images, except for the middle image (which represents 50/50% face features from source image 1 and source image 2) as there is no correct identification of this image. The different face images were varied trial-by-trial according to participants' responses using a staircase procedure. Here, we manipulated the difficulty levels in terms of face discrimination difficulty by moving along the morph continuum (consequently varying the weight of face features from the two source images). The levels of difficulty here was each image on the continuum was defined as the “distance” in image steps from the midline image (which is 50/50% of the two source images). For example both Image 5 and Image 27 on the morph continuum are equidistant from the midline and therefore represent the same level of difficulty−11 (there are 11 intermediate morphs between the midline and both Image 5 and Image 27). Indeed, in both images the makeup of face features is 86.67 and 13.33%. Overall, this resulted in 15 morph levels (and therefore 15 different difficulty levels of discrimination). For the purpose of establishing a threshold metric, large values represent large differences (and therefore easier conditions) while small values reflect small differences (the image is closer to the 50/50% midline point; see Figure 2). Here too the opacity of face stimuli was kept constant at 0.2 throughout.

###### 2.3.1.3.2 Procedure

In each trial, participants were required to make two alternative forced choice responses on whether a face target stimulus is more similar to one source image (source image 1) or to the other (source image 2). The display sequence and the response keys were identical to the ones in the face-noise task. The task procedure with respect to threshold estimation was similar to the face-noise task (in terms of the two interleaved staircases). However, here performance threshold was measured as the position on the morph continuum. Thus, the 3/1 staircase procedure changed the position on the continuum (the distance from the 50/50% on the continuum in either direction) by one place: change following an error moved the point one place away from the 50/50% point, and change following three consecutive correct responses moved the position one place closer to the 50/50% point. The two starting positions (for the interleaved staircases) were 6 and 11 on the continuum, which represent difficult (closer to the 50/50% point) and easy (further away from the 50/50% point) levels of discrimination, respectively. The step size was constant for all reversals (1 place on the morph continuum). Thus, the threshold estimates were given as the position on the morph continuum (or morph distance). As before, threshold estimates were calculated by averaging the reversals on each staircase separately after excluding the first three reversals and then averaging across all staircases across all blocks. Similar to the noise tasks, low values of this threshold estimate reflect better perceptual performance.

#### 2.3.2 Gabor-noise task

##### 2.3.2.1 Stimuli

The Gabor stimuli (3° × 3° visual angles) were presented with a spatial frequency of 1.5 cycles per degree, orientated either at 54° or 36° (±9° from 45 degrees) and embedded in additive Gaussian noise of mean value 0 and SD of 0.33. The opacity of the Gabor stimuli was kept constant at 0.1 throughout. Weber Contrast is 0.12. In contrast to previous studies (Dosher and Lu, 2005), we used foveal Gabor stimuli (see Figure 3) instead of peripheral to reduce the impact of attention processes (particularly dividing attention) and to keep the noise in a similar position as in the face-noise task.

##### 2.3.2.2 Procedure

Participants discriminated the orientation of the Gabor patches with reference to a standard orientation of 45°. The standard orientation was never shown. Participants were instructed to identify the orientation of the Gabor patch by pressing the left or the right arrow keys on the keyboard (two alternative forced choice responses). The general procedure, including the psychophysical staircase procedure of the noise opacity, was identical to the one used in the face-noise task, with two changes: (1) Gabor stimuli were shown for 41.65 ms instead of 66.64, and (2) the two interleaved staircases started at value of 0.45 and at value of 0.35 (instead of 0.85 and 0.75, respectively). The threshold estimates were calculated in the same way as those for the face-noise task.

### 2.4 General procedure

The experiment consisted of three phases: pre-training (first day), training (days 2 through 4) and post-training (day 5). These add up to 5 days of testing and training. We have opted to use this design with three training days in between pre- and post-training days to align with the protocol used by Chang et al. (2013), which is a crucial study in the context of the learning transfer effects we were investigating here. All 28 participants from the three training groups performed the training phase within three successive days. Twenty-six participants completed the entire experiment within five successive days while one participant from the face-feature task training group and one participant from the Gabor-noise task training group completed the study within 6 and 8 days, respectively. In the control group, where training was not conducted, participants completed the experiment (pre-training and post-training sessions only) within 5 or 6 days (from their first testing session to their final testing session inclusively).

For the pre-training and post-training sessions, participants performed three blocks of 120 trials (360 trials in total) on each of the two noise tasks—face-noise and Gabor-noise. The order of these noise tasks was counter-balanced across participants at the pre-training session and was maintained at the post training session. For each of the three training days, participants performed five blocks of 120 trials (600 trials in total) of the relevant training task according to their group affiliation.

At the beginning of each task, participants were provided with written instructions. Each task started with a short practice session (10 trials), when visual feedback (a small white central asterisk) was presented for 400 ms after correct responses. The practice session was repeated if the rate of errors exceeded 30%. Each practice task was similar to its experimental task in terms of the general procedure, including the psychophysical staircase procedure. In each task, participants were required to respond as accurately as possible and at a comfortable speed. No feedback was provided during the experimental blocks.

### 2.5 Face familiarization procedure

Prior to the pre-training session on the first day, participants participated in a preparation stage designed to familiarize them with the face images. The face images we used were unfamiliar faces; therefore, we first exposed our participants to these faces to make sure they could recognize them. This was done both with the orignal images and the morphed ones as follows.

The preparation stage was composed of two sub-tasks which were administered in a fixed order: identification of two source images (Figure 2, top), and then identification of two experimental images (Figure 2, top). Each sub-task consisted of one block of 20 trials. In each trial a single face image was located in the center of the screen, and participants were required to respond “left” for source/experimental image 1 or “right” for source/experimental image 2. Both face images on each sub-task were presented in random order. The stimulus was presented for 66.64 ms, preceded by a 250 ms black central fixation point and separated from it by a blank interval of 300 ms. The time for response was unlimited. The inter-trial interval (ITI) was 900 ms, including a visual feedback (a small white central asterisk) that was given for 400 ms after an incorrect response. Accuracy rate for each trial was recorded. The two sub-tasks were repeated if the rate of errors had exceeded 20% on the last 10 trials. Participants were excluded from further participation in the study if they failed to recognize these faces (errors exceeding 20% after two practice runs). A total of nine participants were excluded from further participation following this stage: seven failed to discriminate between the two source faces and two failed to discriminate accurately between the experimental faces.

### 2.6 Data reduction

In order assess learning transfer effects we wanted to make sure participants in the analysis show learning. Thus, to avoid contaminating the data with participants who do not show learning effects participants were excluded from further analysis if they failed to demonstrate learning. To define a robust criterion that could be applied across all the different tasks and procedures in our study we defined this as a failure to improve performance from the first to the second day the perceptual task was performed. This resulted in the exclusion of nine participants, as follows: Eight participants (one from the Gabor-noise task training group and seven from the face-noise task training group) were excluded due to no improvement between the pre-training session and the first day of training. An additional participant did not show improvement from the first to the second day of training in the face-feature task training group and was also excluded. The main data analyses were repeated with the excluded nine participants, resulting in a similar pattern of findings but with marginal significance levels using parametric tests and similar significant results using a-parametric tests (see Supplementary Tables S1, S2).

### 2.7 Data analysis

To assess the effects of perceptual learning we analyzed the discrimination thresholds scores in the two noise tasks: faces and Gabor, before and after training across the four groups by using a mixed model ANOVA analysis. The data is presented as mean with Standard Error (SE). Effect sizes are given as partial eta squared (partial η*2*) for ANOVA and Cohen's *D* for simple effects. Furthermore, a learning curve, representing discrimination threshold scores as a function of the time course of training, was fitted with a power function and evaluated by regression analysis for each training group separately.

## 3 Results

**Figure 4:**
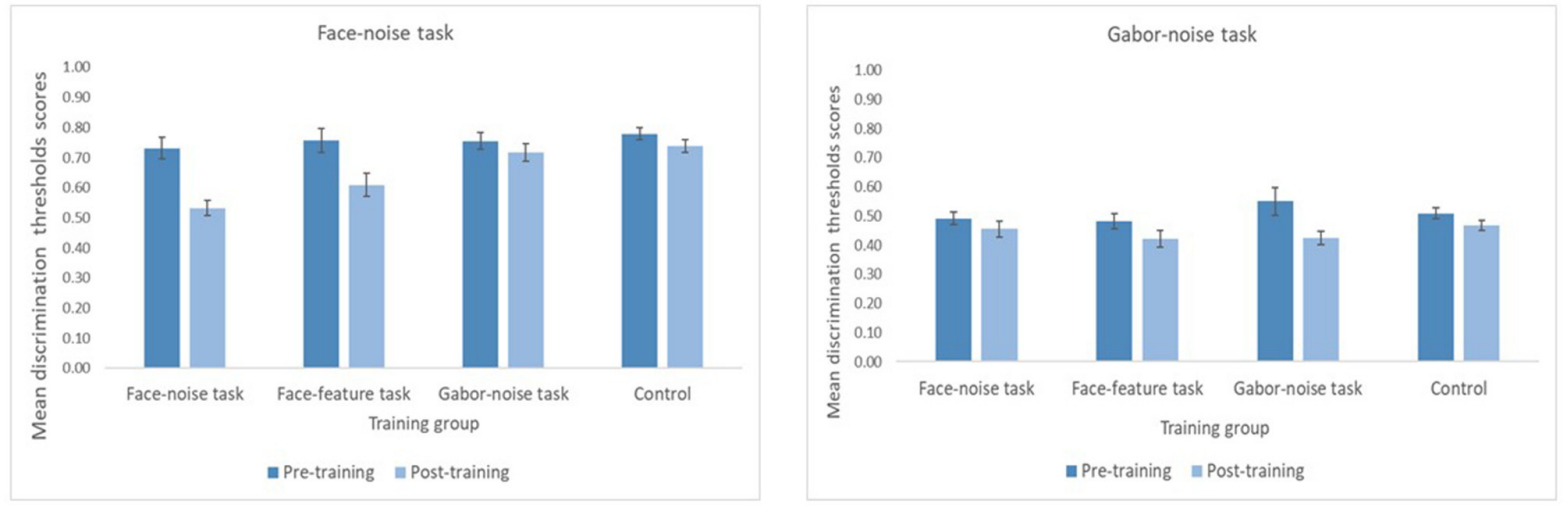
Noise threshold estimates before and after training for the two noise tasks, separately for each of the four training groups. Lower thresholds represent better performance. Threshold estimates were calculated as 1opacity of the noise. Error bars represent ± standard error.

To examine learning and transfer of learning in feature discrimination and noise tasks, we measured thresholds for the face-noise task before and after a period of training on either the face-noise task, the Gabor-noise task, the face-feature task or the control group (no training). First, we focus on the question of transfer of learning from feature learning to noisy displays within complex visual stimuli (faces). We analyzed these data using a mixed model ANOVA with training group (face-noise, Gabor-noise, face-feature, Control) as a between subject factor and timing of testing (pre-training vs. post-training) as the within subject factor (see Figure 4). We found a significant main effect of timing, *F*_(1, 33)_ = 54.00, *p* < 0.0001, η*2* = 0.62, with a lower threshold (better performance) after training (*M* = 0.65, *SE* = 0.01) than before it (*M* = 0.76, *SE* = 0.02). More importantly, we observed a significant two-way interaction of type of training and timing of testing, *F*_(3, 33)_ = 7.52, *p* < 0.001, η*2* = 0.4). Simple effects revealed that prior to training, threshold estimates in the face-noise task were comparable across the four training groups, *F*_(3, 33)_ = 0.36, *p* = 0.78, while significant differences in threshold estimates were observed after training, *F*_(3, 33)_ = 10.50, *p* < 0.0001, η*2* =0.49. We have also conducted non-parametric tests over the data and the results remain the same (see Supplementary Table S3). Specifically, threshold estimates on the face-noise task were similar following training on both the face-noise task and face-feature task (Tukey's test: *p* = 0.26), and were both significantly better than for the control group (Tukey's test: *p* < 0.0001, *p* < 0.05, respectively). In contrast, threshold estimates following training with the Gabor-noise task did not differ from the control group (Tukey's test: *p* = 0.96) but were significantly worse than the face-noise task (Tukey's test: *p* < 0.001).

Comparing between the two time points—pre-training and post-training for each training group separately—revealed the same pattern of results. Specifically, participants improved their performance after training in the following groups: face-noise training [*t*_(8)_ = 7.295, *p* = < 0.01], face-feature training [*t*_(9)_ = 3.522, *p* = < 0.01]. Participants from the control group also showed significant improvement in the face-noise task [*t*_(8)_ = 2.479, *p* = < 0.05]. In contrast, the improvement of participants from the Gabor-noise training group did not reach statistical significance [*t*_(8)_ = 2.087, *p* = 0.07].

The above analysis suggests that improvement in the face-noise task was more pronounced in the group who trained with the face-noise and with face-feature task, compared to the Gabor-noise and control. To further ascertain the changes in performance in the face-noise task, we quantified the improvement between the two time points (ratio of change relative to baseline performance). This improvement index (given as a percentage) was calculated as follows:


$$\begin{array}{l} \text{Improvement index}=\frac{Pre\;Training-\;Post\;Training}{Pre\;Training}*100 \end{array}$$

The difference in discrimination estimates in the two time point (pre training *minus* post training) divided by the pre-training estimate (and then multiplied by 100 to give percentage). This measure eliminates differences that may have occurred at baseline (although no statistical difference was found between the groups in their baseline performance). For the control group, the magnitude of improvement in the face-noise task was 5.21%, which reflects a baseline improvement expected without any training (i.e., as a result of individuals' familiarity with the face-noise task). We therefore used this measure as a baseline for improvement against which the training groups could be compared through the statistical Dunnett's test and found significant difference between the training groups, *F*_(3, 33)_ = 8.18, *p* < 0.0001, η*2* = 0.43. Specifically, participants who trained on both the face-noise task and the face-feature tasks showed significantly higher improvement compared to the control group (improvement index = 26.65%, *p* < 0.001; improvement index = 18.48%, *p* < 0.05, respectively). There was no significant difference in the magnitude of improvement between these two training groups, *t*_(17)_ = −1.27, *p* = 0.22, and between participants who trained on the Gabor-noise task and the control group (improvement index = 4.76%). Parallel non-parametric tests revealed similar effects (see Supplementary Table S4).

Next, we quantified the effect of learning across groups by calculating a transfer index (see Chang et al., 2013) using the improvement index described above as follows.


$$\begin{array}{l} \text{Transfer index}=\frac{\text{Between}}{\text{Within}} \end{array}$$

The improvement index in one of the groups who did not train with the face-noise task (i.e., the face-feature task and Gabor-noise task; between task improvement) divided by the improvement observed in the group training with the face-noise task (within task improvement). A value of 1 expresses a similar improvement between and within (a full transfer of learning). The results indicated that training on the face-feature task led to improvement on the face-noise task, with approximately 70% transfer, whereas the training on the Gabor-noise task showed similar changes to participants who did not train (transfer indices of 18 and 19%, respectively).

**Figure 5:**
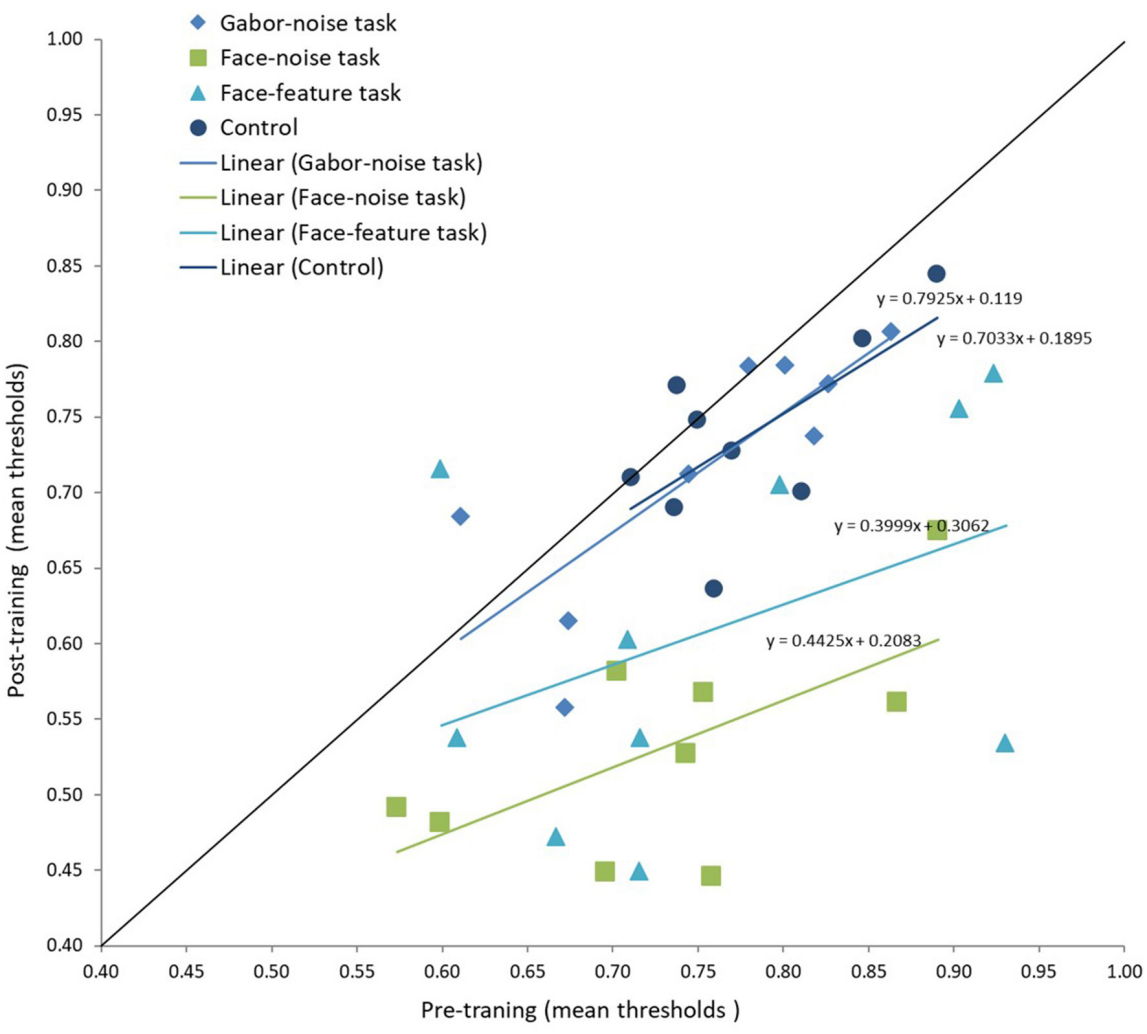
Visualization of the magnitude of improvement on the face-noise task in the four training groups. The threshold estimates are presented for each participant for the pre (x-axis) and post (y-axis) training sessions. The slopes, which reflect the degree of learning, were calculated across participants from the same training group. A slope value close to 1 (the diagonal) represents limited learning, while a shallow slope represents more substantial learning and improvement.

To further visualize the learning effects in the face-noise task in each training group, we plotted participants' performance on the face-noise task before and after the training session and fitted a regression line for each group separately (see Chang et al., 2013; Figure 5). For the Gabor-noise training group and the control group the slope parameter of the regression line is indistinguishable from 1 (*B* = 0.79; *B* = 0.70, respectively). A slope of unity (displayed as a diagonal reference line in Figure 5) represents equal performance in terms of discrimination thresholds in the pre- and post-training sessions, and thus reflects no learning. In contrast, for both the face-noise and face-feature training groups, the slope parameter of the regression line was substantially different than the diagonal reference line (*B* = 0.44; *B* = 0.40, respectively*)*, indicating a high learning of the face-noise and transfer of learning between the face-feature and face-noise tasks (see Figure 5).

**Figure 6:**
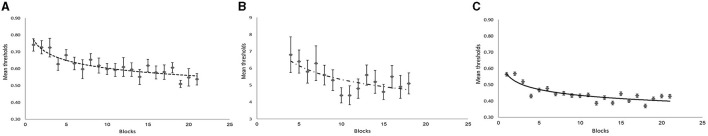
Learning curve for each training group. Power function was fitted to discrimination threshold on pre-training (blocks 13), training (blocks 418), and post-training (blocks 1921) sessions for participants trained on face-noise task **(A)** and those trained on Gabor-noise task **(C)**. Power function was fitted to discrimination thresholds from the training session only (blocks 115) for participants trained on face-feature task **(B)**. Error bars represent ± standard error.

A further consideration was assessing the extent of learning throughout the course of training (learning curve) in the active training groups (face-noise group and face-feature training). These groups showed improvement, which manifested in a gradual decrease in thresholds throughout the training process (Figure 6). Particularly, the face-noise group demonstrated a significant decrease in threshold estimates from 0.74 (SE = 0.04) in block 1 to 0.54 (SE = 0.03) in block 21, *F*_(1, 19)_ = 69.97, *p* < 0.001, Adjusted *r*^2^ = 0.75 (*SE* = 0.05) (Figure 6A). The face-feature group also demonstrated a significant decrease in thresholds from 6.8 (SE = 1.06) in block 1 to 5.10 (SE = 0.62) in block 15, *F* (_1, 13)_ = 12.92, *p* < 0.01, Adjusted *r*^2^ = 0.46 (*SE* = 0.10) (Figure 6B). On the face-feature task, power function was fitted only to 15 training blocks (blocks 4–18). The pre-training (blocks 1–3) and post-training (blocks 19–21) section did not include this type of task.

To exclude the possibility that the reason we found no transfer effects in the Gabor-noise training group is because no learning was achieved in this training group (in the Gabor-noise task itself), we also fitted a learning curve to the threshold estimates in the Gabor-noise task. However, we found that the Gabor-noise group did demonstrate a significant decrease in thresholds from 0.56 (SE = 0.06) in block 1–0.43 (SE = 0.03) in block 21, *F*_(1, 19)_ = 50.10, *p* < 0.001, Adjusted *r*^2^ = 0.71 with *SE* of 0.06 (Figure 6C). Thus, participants in the Gabor-noise training group did demonstrate perceptual learning in the trained task.

Finally, we also considered the magnitude of learning on the Gabor-noise task across the different training tasks to quantify learning and possible transfer effects on the Gabor-noise. We analyzed these data using a mixed model ANOVA with type of training group as a between subject factor (face-noise, Gabor-noise, face-feature, Control) and timing of testing (pre-training vs. post-training) as the within subject factor (see Figure 4). We first confirmed there was no statistical difference in threshold estimates prior to training across the four groups of participants, *F*_(3, 33)_ = 0.998, *p* = 0.41, η*2* = 0.08. We found a significant main effect of timing, *F*_(1, 33)_ = 24.75, *p* < 0.0001, η*2* = 0.43: with a lower threshold (better performance) after training (*M* = 0.44, *SE* = 0.01) than before (*M* = 0.51, *SE* = 0.02). However, there were no significant group differences, *F*_(3, 33)_ = 0.50, *p* = 0.68, η*2* = 0.04 and the interaction between training group and timing of testing was not significant, *F*_(3, 33)_ = 2.39, *p* = 0.087, η*2* = 0.18 (see Figure 4). We have also conducted non-parametric tests over the data and the results remain the same (see Supplementary material). Thus, for the Gabor-noise task, we could not ascertain learning or transfer effects that are over and above what can be documented by a simple repetition of the Gabor-noise (e.g., the control group).

## 4 Discussion

The main aim of the current study was to examine the previously documented feature-to-noise perceptual learning transfer effect (Dosher and Lu, 2005; Chang et al., 2013) in the context of face stimuli. We therefore estimated noise thresholds of face stimuli embedded in noise (face-noise) before and after training in four different training protocols. Critically, we included both perceptual learning using the same face-noise task and perceptual learning in a face-feature task (without noise) as well as a control group where no training took place. Our data indicate a substantial transfer effect from the face-feature task to the face-noise task, which provides initial evidence that feature-to-noise transfer effects are not limited to simple perceptual judgment and can occur with complex real-life stimuli too. A secondary (exploratory) aim of the current study was to examine possible transfer effect across different stimuli in noise training. In particular, we assessed whether perceptual learning of a simple perceptual judgment (orientation) embedded in noise will transfer to complex perceptual discrimination (faces) embedded in similar type of noise. However, we found no evidence for such transfer using the current design and parameters.

Our implementation of four separate groups for the training stage, enabled us to safely identify learning and transfer effects over and above improvement in performance that may be shown in the control group (simply associated with task repetition). We first established that there were no differences in baseline performance between the training groups and we then measured performance differences following training both in absolute (threshold estimate in the post-training session) and relative (% change in performance in the post- vs. pre-training session) terms. In the face-noise task, we observed significant transfer effects. Notably, we detected no statistical difference in the extent of improvement between the face-noise training group and face-feature training group. Training on the face-feature task resulted in transfer equivalent to 70% of the learning magnitude observed in the face-noise task, representing a substantial transfer, especially when compared to the 19% of spontaneous learning seen in the control group. Conversely, training in the Gabor-noise task did not yield improvement or transfer to the face-noise task beyond the spontaneous improvement demonstrated by the control group.

The transfer effects we report are aligned with previous studies documenting transfer from clear to noisy tasks on simple visual features (Dosher and Lu, 2005; Chang et al., 2013). Dosher and Lu (2005) interpreted such results of learning under clear and noisy conditions as two independent processes of the perceptual system: (1) Segmentation of the visual feature (or noise filtering); and (2) feature representation. Training in clear tasks improves internal feature representation and is used in both clear and noisy tasks, whereas training in noise tasks impacts only the noise filtering mechanism. Using brain stimulation, Chang et al. (2014) demonstrated a double dissociation between these processes with noise filtering (in a depth perception in noise task) dependent on left parietal cortex (Mevorach et al., 2010) while feature representation (in a feature discrimination depth perception task) implemented in the lateral occipital area (LO). Critically however, perceptual learning with a feature depth discrimination task changed the contribution of the parietal and LO areas in the depth noise task so that after training performance only depended on LO. Thus, the authors concluded the feature-to-noise transfer was a consequence of feature representation processes in LO supplanting noise filtering processes in the parietal cortex (Chang et al., 2014). Our findings here suggest that these processes are not limited to simple perceptual features and may be involved when complex images, such as faces, are judged.

Another explanation for the feature-to-noise transfer refers to the different learning efficiencies (Lu et al., 2010). Lu et al. (2010) applied the augmented Hebbian re-weighting model and tested it on previous perceptual learning experiments with external noise (e.g., Dosher and Lu, 1998, 1999, 2005). According to this model, performance improvement occurs as the system gradually allocates higher weight to the relevant features and lower weight to irrelevant features. Different training conditions (clear or noisy) impact the locus of the adjusted weight allocation. Accordingly, the asymmetry in transfer is a consequence of different adjustment of weighting in the clear and noisy conditions with the former leading to optimal use of a relevant feature over the irrelevant ones and the latter leading to decreases in the weight given to the irrelevant features. Thus, training in high-noise cannot lead to optimal weight allocation to the relevant features because it is not “learned” in the high-noise condition (Lu et al., 2010). Although our failure to induce transfer effects between two (very) different noise tasks should be taken with caution (see below), it may suggest that such reweighting of noise attributes is still dependent on the target stimulus, or the combination of noise and signal, and therefore is not transferable even when the noise itself is repeated across different tasks.

The transfer effects we report here with complex stimuli also align with previous reports demonstrating transfer with complex images. For instance, Baeck and Op de Beeck (2010) have demonstrated transfer effects when using the same objects under different perceptual protocols. In fact, the authors reported that masked object identification partially transferred to conditions where the same object was presented together with noise. Our findings of transfer from the face-feature task (without noise) to the face-noise task can be taken together with such previous findings to support the notion that the basic finding reported by Dosher and Lu (2005) with simple visual stimuli, can be replicated in complex visual stimuli too. However, in the current study the transfer effect is shown not across identical objects but rather across the ability to distinguish between two faces using different degrees of similarity (or distance). Our face-feature training was designed in a way that will promote learning of feature representation through increasing the precision. This was then shown to help participants distinguish between faces needing less precision but embedded in external noise.

Previous studies have argued that specificity and transfer in perceptual learning depend on the difficulty or precision of the training task (Ahissar and Hochstein, 1997, 2004; Liu and Weinshall, 2000; Wang, 2013; DeLoss et al., 2014). This aspect may be particularly pertinent to our study, considering the challenge posed by the face identification task exemplified by the relatively high rate of participant who could not learn to discriminate between the faces. Ahissar and Hochstein (1997, 2004), for instance, used a simple visual detection task (report the presence or absence of an oddly oriented bar in an array of oriented distractors bars) while manipulating task difficulty and found that specificity increased with increased difficulty. Although the context of a feature-to-noise transfer is somewhat different, according to this account we would expect a transfer from the face-feature to the face-noise task if the face-feature task represents an easier learning task. While we cannot ascertain this here, it is worth noting that a higher proportion of participants in our study failed to show learning effects in the face-noise task than the face-feature task, which may fit with this idea.

However, a different account which may better fit our findings suggests that specificity and transfer effects are related to the tested rather than trained task (Jeter et al., 2009). Jeter et al. (2009) used an orientation discrimination task under two different precision demands. Their findings demonstrated that when testing on the lower precision task similar (and substantial) transfer effects were evident after training with both the low and high precision versions. In contrast, no transfer was evident when testing on the higher precision task. In our study, the face-noise task required lower precision of face discrimination (the faces were located further apart on the morph continuum). It could therefore be argued that here too, lower precision demands of the tested task led to transfer effects (including using a higher precision task for training).

In contrast to the feature-noise transfer effect, we did not find evidence for transfer across the two noise tasks, as the Gabor-noise group showed similar improvements as the controls. Indeed, this lack of transfer was also demonstrated in the opposite direction with the face-noise group showing similar improvement in the Gabor-noise as controls. These findings, therefore, fail to provide support to previous reports suggesting that noise filtering could be trained in perceptual learning and could be subsequently implemented even when the specific feature that needs to be discriminated is changed (Chang et al., 2013). One possibility here is that perceptual learning involving noise filtering cannot be transferred in the context of complex stimuli. This is supported by a previous attempt using complex objects in noise displays that also failed to show transfer to non-trained objects (Baeck and Op de Beeck, 2010). However, there are other more likely possibilities that reflect the specific parameters we have used. First, it is possible that the lack of transfer we report was due to change in stimuli complexity between the Gabor-noise and face-noise task. In Chang et al. (2013), the noise filtering transfer effects occurred between different tasks using simple features (motion direction, orientation and depth). In contrast, in our study we tested the transfer from simple feature (orientation) to complex features (face identity) and vice versa. It is therefore plausible that transfer could only occur when there is similarity in the levels of target feature complexity (although see Baeck and Op de Beeck, 2010). Secondly, and perhaps more likely, our implementation of the Gabor-noise task may have driven this lack of transfer: the Gabor patches were unusually centrally presented and showed limited evidence for learning (we found no significant group differences across the different training groups in the Gabor-noise task). While learning did occur (demonstrated using a learning function) it may have been confounded by a ceiling effect. Indeed we also found a substantial difference at baseline (pre-training) between threshold estimates of the Gabor and face-noise tasks with considerably lower threshold for the Gabor task (Gabor noise task; *M* = 0.51, *SE* = 0.02 compared to face-noise task; *M* = 0.76, *SE* = 0.02). We, therefore, conclude that the learning observed in the Gabor-noise task and its potential transfer effects should be interpreted with caution.

The transfer effect we report from the face-feature training task to the face-noise task is particularly interesting as it suggests that individuals who may exhibit difficulty in filtering noise might benefit from training in a fine-discrimination (no-noise) task. Performance in tasks with external noise is thought to rely on attention processes of noise filtering (e.g., Chang et al., 2014). It is therefore possible that attention impairment may be related to difficulties in noisy tasks or deficient learning in noisy tasks, but that training with feature discrimination provides an alternative route for improvement.

## 5 Limitations

This study has a few limitations. First our sample size was relatively small although not unlike previous similar studies (e.g., Jeter et al., 2009; Bi et al., 2010; Chang et al., 2013). Second, our use of a difficult face discrimination task may limit our findings to the context of a specific (special) category of complex stimuli. Moreover, the difficulty of the face discrimination task led to the exclusion of participants who could not accurately identify the faces or demonstrate learning. This therefore limits our results to a sub-set of the population which is less likely to experience face processing difficulties. Using more easily discernible faces or even familiar faces may lead to a reduction in the number of participants excluded from the study and to a more generalizable participants cohort. Moreover, in the present study, participants underwent only 3 days of training. Extending the training period could potentially enhance the intensity of learning, and this aspect should be taken into account in future research. Finally, there was a marked difference in the current study between the Face noise and Gabor noise tasks, both in terms of the overall difficulty (with higher thresholds for the face noise task) and in the magnitude of learning (with no significant difference over the control for the Gabor noise task). As such, we were unable to ascertain whether noise filtering could have been transferred across the two tasks. Future studies should attempt to equate these aspects between the tested signal-in-noise tasks.

## Data availability statement

The raw data supporting the conclusions of this article will be made available by the authors, without undue reservation.

## Ethics statement

The studies involving humans were approved by Ethics Committee of Tel Aviv University. The studies were conducted in accordance with the local legislation and institutional requirements. The participants provided their written informed consent to participate in this study.

## Author contributions

OA: Conceptualization, Methodology, Data curation, Project administration, Writing – original draft. LS: Conceptualization, Supervision, Writing – review & editing. CM: Conceptualization, Methodology, Supervision, Writing – review & editing.
