# Supplementary material for: Feature discrimination learning transfers to noisy displays in complex stimuli
Source: Front Cognit. 2024 Mar 6;3:1349505. doi: 10.3389/fcogn.2024.1349505 (PMC13281115; doi:10.3389/fcogn.2024.1349505)
Supplement: Supplementary file 1 [file Table_1.DOCX]

**Supporting Information**

**Feature discrimination learning transfers to noisy displays in complex stimuli**

Orly Azulai [1], Lilach Shalev [1, 2], & Carmel Mevorach [3,4]

[1] *School of Education, Tel Aviv University, Israel*

[2] *The Sagol School of Neuroscience, Tel Aviv University, Israel*

[3] *School of Psychology, University of Birmingham, UK*

[4] *Centre for Human Brain Health, University of Birmingham, UK.*

Corresponding author:

Orly Azulai
Tel Aviv University, Israel

Orli.azulai@gmail.com

1. **Extended data (including non-learners) face-noise**

**1.1 Parametric analysis**

Means and SDs on the face-noise task for the 46 participants (including learners and non-learners) are presented in Table S1.

**Table S1:** Performance on the face-noise task by training groups

| Training groups |  | pre-training session | post-training session | improvement index |
| --- | --- | --- | --- | --- |
| Face-noise task | 16 | 0.684 | 0.562 | 16.734 |
|  |  | (0.116) | (0.100) | (14.086) |
| Face-feature task | 11 | 0.739 | 0.603 | 17.118 |
|  |  | (0.133) | (0.116) | (16.884) |
| Gabor-noise task | 10 | 0.765 | 0.714 | 6.290 |
|  |  | (0.087) | (0.080) | (8.970) |
| Control | 9 | 0.779 | 0.737 | 5.213 |
|  |  | (0.059) | (0.063) | (6.502) |

The repeated measure ANOVA with time (pre vs. post training) as within subject factor and training group (face-noise, face-feature, Gabor-noise and control) as between subject factor, revealed a main effect for time of testing (F (1, 42) = 33.65, *p* < .001, η2 = .44). The interaction between time and group was marginally significant (F (3, 42) = 2.51, p = .072, η2 = .152).

**1.2 Non-parametric analysis**

The non-parametric analysis on the improvement index in the face-noise task revealed a significant main effect of group, Kruskal-Wallis test H(3)=9.42, *p* < .05. Multiple pairwise comparisons between training groups are presented in Table S2.

**Table S2:** Improvement indices in the face-noise task: Pairwise comparisons between groups

|  | Dunn’s pairwise tests | *p* |
| --- | --- | --- |
| Face-noise-control | 13.05 | < .05 |
| Face-feature-control | 14.47 | < .05 |
| Gabor-noise-control | 2.81 | 0.65 |
| Face-noise-Face-feature | 1.43 | 0.78 |
| Face-noise-Gabor-noise | 10.24 | 0.058 |
| Face-feature-Gabor-noise | 11.66 | < .05 |

**2. Original data face-noise – Non-parametric analysis**

**2.1 Pre and post training sessions:** Performance on the face-noise task before training was similar in the four groups, Kruskal-Wallis test H(3)= 1.28, *p* = .73. In contrast, there was a significant main effect of groups after training: Kruskal-Wallis test H(3) = 16.56 p < .001. Multiple pairwise comparisons between training groups are presented in Table S3.

**Table S3:** Improvement indices of the face-noise task: Pairwise comparisons between groups

|  | Dunn’s pairwise tests | *p* |
| --- | --- | --- |
| Face-noise-control | -17.55 | < .01 |
| Face-feature-control | -10.73 | < .05 |
| Gabor-noise-control | -0.66 | 0.89 |
| Face-noise-Face-feature | 6.82 | 0.17 |
| Face-noise-Gabor-noise | -16.88 | < .001 |
| Face-feature-Gabor-noise | -10.06 | < .05 |

**2.2 Improvement scores:** The non-parametric test of the *improvement indices* revealed a significant main effect of group, Kruskal-Wallis test H(3)=19.096, *p* < . 001. Multiple comparisons for the four training groups are presented in Table S4.

**Table S4:** Improvement indices of the face-noise task: Pairwise comparisons between groups

|  | Dunn’s pairwise tests | *p* |
| --- | --- | --- |
| Face-noise-control | 18.33 | < .001 |
| Face-feature-control | 12.81 | < .01 |
| Gabor-noise-control | 0.78 | 0.88 |
| Face-noise-Face-feature | -5.52 | 0.27 |
| Face-noise-Gabor-noise | 17.56 | < .001 |
| Face-feature-Gabor-noise | 12.03 | < .05 |

**3. Original data Gabor-noise – Non-parametric**

**Pre and post training sessions:** Performance on the Gabor-noise task before and after the training was similar in the four groups, pre-training: Kruskal-Wallis test H(3)= 2.0, *p* = .57; post-training: Kruskal-Wallis test H(3)= 4.40, *p* = .22.
